# *PPARG* in Human Adipogenesis: Differential Contribution of Canonical Transcripts and Dominant Negative Isoforms

**DOI:** 10.1155/2014/537865

**Published:** 2014-03-23

**Authors:** M. Aprile, M. R. Ambrosio, V. D'Esposito, F. Beguinot, P. Formisano, V. Costa, A. Ciccodicola

**Affiliations:** ^1^Institute of Genetics and Biophysics “Adriano Buzzati-Traverso”, National Research Council, 80131 Naples, Italy; ^2^Department of Translational Medical Sciences, University of Naples “Federico II”, 80131 Naples, Italy; ^3^Institute of Experimental Endocrinology and Oncology, National Research Council, 80131 Naples, Italy

## Abstract

The nuclear receptor PPAR**γ** is a key regulator of adipogenesis, and alterations of its function are associated with different pathological processes related to metabolic syndrome. We recently identified two *PPARG* transcripts encoding dominant negative PPAR**γ** isoforms. The existence of different *PPARG* variants suggests that alternative splicing is crucial to modulate PPAR**γ** function, underlying some underestimated aspects of its regulation. Here we investigate *PPARG* expression in different tissues and cells affected in metabolic syndrome and, in particular, during adipocyte differentiation of human mesenchymal stem cells. We defined the transcript-specific expression pattern of *PPARG* variants encoding both canonical and dominant negative isoforms and identified a novel *PPARG* transcript, **γ**1ORF4. Our analysis indicated that, during adipogenesis, the transcription of alternative *PPARG* variants is regulated in a time-specific manner through differential usage of distinct promoters. In addition, our analysis describes—for the first time—the differential contribution of three ORF4 variants to this process, suggesting a still unexplored role for these dominant negative isoforms during adipogenesis. Therefore, our results highlight crucial aspects of *PPARG* regulation, suggesting the need of further investigation to rule out the differential impact of all *PPARG* transcripts in both physiologic and pathologic conditions, such as metabolism-related disorders.

## 1. Introduction 

Peroxisome proliferator-activated receptors (PPARs, also known as nuclear receptor family 1C, NR1C) are ligand-dependent transcription factors belonging to the nuclear hormone receptor superfamily. Three members of the PPAR family—known as PPAR*α*, PPAR*β*/*δ*, and PPAR*γ*—encoded by different genes located on different chromosomes have been identified [1–3].

Undoubtedly, PPAR*γ* is the most extensively studied and characterized member of PPARs, given its involvement in several physiological states, as well as pathological conditions. Indeed, it modulates the expression of several genes that play a central role in glucose, lipid and cholesterol metabolism, inflammation, angiogenesis, proliferation, and differentiation [4–7]. In particular, PPAR*γ* is the master regulator of adipogenesis, since it regulates the transcription of a wide number of genes involved in cellular differentiation and lipid accumulation [8, 9]. Defects in PPAR*γ*, signaling its altered expression and/or activation, as well as polymorphisms/mutations, are implicated in different pathological conditions occurring in metabolic syndrome, such as insulin resistance, obesity [10], dyslipidemia, and hypertension, that markedly increase the risk of type 2 diabetes [11–13], as well as cardiovascular diseases and cancer [3, 4, 14–17].

The prevalence of metabolic syndrome is increasing to epidemic proportions and, to date, an adequate therapy has not been yet established. Of great clinical interest, synthetic ligands of PPAR*γ*, belonging to the class of thiazolidinediones (TZDs), such as troglitazone, pioglitazone, and rosiglitazone, function as insulin sensitizers and are used for treating hyperglycemia in patients with type 2 diabetes [7, 18–20]. Nevertheless, their use in type 2 diabetes therapy has been limited by untoward effects. Thus, a better understanding of PPAR*γ* signaling is crucial to develop more effective and targeted therapeutic strategies to treat metabolic syndrome and its complications.

However, to fully define the landscape of PPAR*γ* activity, some relevant aspects need to be taken into account. One of the most relevant features is the ability of* PPARG* gene to give rise to different transcripts. Indeed, the human* PPARG* gene consists of nine exons and—by differential promoter's usage and alternative splicing—generates at least four main splice variants (i.e., PPARG1, PPARG2, PPARG3, and PPARG4). These transcripts display different 5′ untranslated regions (UTRs), followed by six coding exons. However, despite the presence of such a variable number of* PPARG* transcripts, this gene encodes only two protein isoforms. Indeed, PPARG1, PPARG3, and PPARG4 encode the same protein PPAR*γ*1—localized in the adipose tissue, liver, heart, and skeletal muscle—whereas PPARG2 yields a protein with 28 additional amino acids at the N-*terminus*, known as PPAR*γ*2, exclusively localized in the adipose tissue [21–23].

Different ability to induce adipogenesis has been shown for PPAR*γ*1 and PPAR*γ*2, indicating a more relevant adipogenic activity for PPAR*γ*2. Although both isoforms are thought to be essential during adipocyte differentiation, their relative contribution is not yet well clarified [24–27].

More recently, our group identified in sporadic colorectal cancers two novel* PPARG* transcripts harboring a read-through in intron 4, named *γ*2ORF4 and *γ*3ORF4, displaying the same 5′UTRs of PPARG2 and PPARG3, respectively [28]. The protein products lack the ligand binding domain (LBD) and act as dominant negative toward PPAR*γ*. Although it has been shown that *γ*ORF4 plays a role in pathogenesis of colorectal cancer, its presence and expression levels have not yet been investigated in other cells and/or tissues.

To date, accurate analyses of the expression pattern of each* PPARG* transcript are still missing. For instance, to the best of our knowledge, this consideration holds true particularly for the adipogenesis, in which PPAR*γ* is the main driver [4, 7, 29]. Alterations of adipocyte differentiation are strictly associated with obesity and metabolism-related disorders and therefore intimately linked to the physiopathology of the metabolic syndrome [30, 31]. Describing in detail the relative contribution of all currently known* PPARG* transcripts—and its dominant negative isoforms—in adipogenesis, as well as in tissues and cells related to processes altered in metabolic syndrome, will provide a solid basis to rule out if, and how, they may account for metabolism-related diseases.

Here we describe a complete expression analysis of all annotated* PPARG *transcripts—PPARG1, PPARG2, PPARG3, and PPARG4—as well as its dominant negative isoform *γ*ORF4 in human tissues and cells affected in metabolic syndrome. In particular, we focus on their differential expression during human adipogenesis, using human mesenchymal stem cells (hMSCs) isolated from the stromal vascular fraction of adipose tissue [32]. After* in vitro* differentiation of hMSCs in adipose cells, by using transcript-specific RT-PCR and Quantitative Real-Time PCR assays, we measured the expression of* PPARG* transcripts at various time points from the induction of adipocyte differentiation, demonstrating the differential contribution of each alternative splice variant. A similar pattern of expression was also observed for total PPAR*γ* and *γ*ORF proteins. In addition, here we describe, for the first time, a novel transcript of* PPARG*, named *γ*1ORF4, similar to the dominant negative *γ*2ORF4 and *γ*3ORF4, previously identified [28]. Finally, we evaluated the abundance of all ORF4 variants during adipocytes' differentiation, also suggesting—for the first time—the involvement of these dominant negative isoforms in human adipogenesis.

## 2. Materials and Methods 

### 2.1. Cell Cultures

Media, sera, and antibiotics for cell culture were from Lonza (Basel, Switzerland). Human Embryonic Kidney 293 cells (HEK239) were cultured in Dulbecco's modified Eagle's medium (DMEM) supplemented with 10% fetal bovine serum (FBS), 2 mmol/L glutamine, 100 units/mL penicillin, and 100 units/mL streptomycin.

Human Mesenchymal Stem Cells (hMSCs) were obtained by abdominal biopsy and cultures established as described previously [33]. The cells were grown in DMEM-F12 (1 : 1) with 10% FBS, 2 mmol/L glutamine, 100 units/mL penicillin, and 100 units/mL streptomycin. Cultures were maintained in a humidified atmosphere of 95% air and 5% CO_2_ at 37°C.

#### 2.1.1. Adipocyte Differentiation

Adipocyte differentiation was achieved as previously described [34]. Briefly, hMSCs were seeded (10,000 cells/cm^2^) and cultured in six-well plates until confluence. Adipocyte differentiation was induced with a differentiation cocktail consisting of 850 nmol/L insulin, 10 *μ*mol/L dexamethasone, 0.5 mmol/L IBMX (isobutylmethylxanthine), 10 *μ*mol/L pioglitazone, 33 *μ*mol/L biotin, and 17 *μ*mol/L pantothenate in DMEM-F12 (1 : 1) supplemented with 3% FBS, 2 mmol/L glutamine, and antibiotics. After 3 days, the medium was changed to a medium containing only insulin and pioglitazone in DMEM-F12 (1 : 1) supplemented with 10% FBS, glutamine, and antibiotics. Culture medium was then changed every 2 days for another 8 days up to obtain a complete adipocyte differentiation of hMSCs. Lipid accumulation was determined by Oil Red O staining as described by Isakson and colleagues [34]. Adipocyte differentiation from hMSCs was performed in triplicate.

### 2.2. RNA Extraction and RT-PCR Assays

Total RNA was isolated from HEK239 and hMSCs at different stages of adipocyte differentiation, using TRIzol solution (Invitrogen) according to the manufacturer's instructions. RNA extracted from the other human tissues, heart, liver, and thyroid, and cells, human colon carcinoma, endothelial progenitor (EPCs), macrophages, and breast cancer (MCF7), employed in our analysis, was obtained in previous studies [28, 33, 35, 36]. For each sample, total RNA (1000 ng) was reverse transcribed using “high-capacity cDNA reverse-transcription kit” (Applied Biosystems, Foster City, CA). cDNAs obtained from human tissues and cells were used as template for RT-PCR assays. PCR amplification with specific primer pairs—designed using Oligo 4.0 and listed in Table 1—was performed using 1 *μ*L of the reverse transcription reaction as template in PCR reactions set up with AmpliTaq Gold (Perkin Elmer). PCR assays have been performed using these amplification conditions: 95°C for 10 minutes, followed by 35 cycles at 95°C for 40 sec, 60°C for 40 sec, 72°C for 30 sec, and 70°C for 7 min. RT-PCR products were of expected length (see Table 1). In each experiment, a sample without reverse transcriptase was used as negative control and it was amplified under the same conditions as the reverse-transcribed RNA.

### 2.3. Cloning and Sequencing

The multiple PCR products (of about 211 and 137 bp, resp.), obtained in RT-PCR assays of PPARG1/PPARG4, have been cloned into Topo Vector II (Invitrogen) according to the manufacturer's instructions. Clones and other RT-PCR products were directly sequenced by Sanger method, confirming the specificity of reactions.

### 2.4. Real-Time PCR

Quantitative Real-Time PCRs were performed on cDNA samples of hMSCs and undifferentiated at different stages of adipocyte differentiation (6 hours, 12 hours, 24 hours, 2 days, 4 days, 7 days, and 10 days after induction of the process). Amplification reaction mix contained 1x SYBR Green PCR master mix (Applied Biosystems), 160 nM of each primer, and 50 ng of cDNA (RNA equivalent) as template. Quantitative Real-Time PCR assays were performed in according to the manufacturer's instructions for the 7900HT Real-Time PCR system (Applied Biosystems) in the same conditions described in [37]. Each assay for the 5 analyzed transcripts was performed in three biological replicates for all the time points. For each cell replicate, Real-Time assays were performed in two duplicated wells. Relative gene expression was measured by using 2^−ΔΔCt^ method. For each assay, expression levels were normalized for the reference values (time point at 0 hours or 6 hours) using glyceraldehyde 3-phosphate dehydrogenase (GAPDH) as housekeeping gene. qRT-PCRs data were reported as mean values and standard deviation of three biological replicates and results analyzed by paired Student *t* test. *P* value <0.05 was considered statistically significant.

### 2.5. Immunoblot Procedure

Total cell lysates were obtained and separated by sodium dodecyl sulfate—polyacrylamide gel electrophoresis (SDS-PAGE) as previously described [38]. Briefly, hMSCs undifferentiated and at different stages of adipocyte differentiation (2 and 10 days) were solubilized for 2 hours at 4°C with lysis buffer containing 50 mM HEPES, 150 mM NaCl, 10 mM EDTA, 10 mM Na_4_P_2_O_7_, 2 mM sodium orthovanadate, 50 mM NaF, 1 mM phenyl-methyl-sulfonyl fluoride, 10 *μ*g/mL aprotinin, 10 *μ*g/mL leupeptin, pH 7.4, and 1% (v/v) Triton X-100 (all reagents for lysis buffer were from Sigma-Aldrich, St Louis, MO, USA). The lysates were clarified by centrifugation at 12,000 rpm for 20 min at 4°C. Proteins were separated by SDS-PAGE (Bio-Rad Hercules, CA, USA) and blotted on Immobilon-P membranes (Millipore, Billerica, MA). Membranes were incubated with a polyclonal antibody directed against the N-terminal domain of PPAR*γ* (Santa Cruz Biotechnology, CA, USA) and with antiactin antibodies (Santa Cruz Biotechnology, CA, USA). Detection of blotted proteins was performed by enhanced chemiluminescence (ECL, Amersham Biosciences, Arlington Heights, IL, USA) according to the manufacturer's instructions. Densitometric analysis was performed using Image Lab Software (Bio-Rad, Hercules, CA, USA). For each protein isoform (PPAR*γ* and *γ*ORF4), data are shown as pixel density ratio versus control protein (actin).

## 3. Results 

### 3.1. Expression Profile of* PPARG* Transcripts

**Figure 1:**
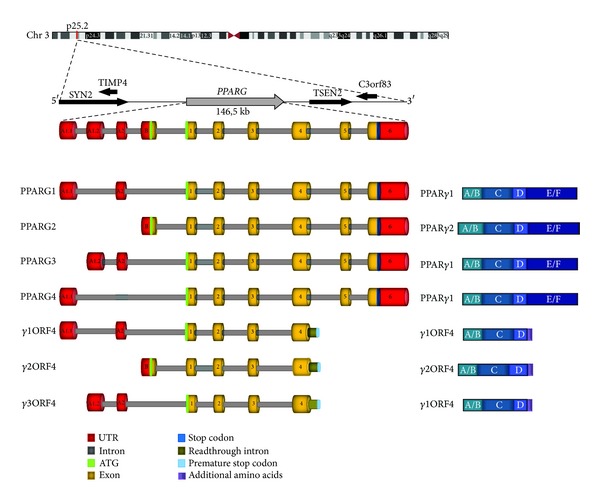
Schematic representation of human* PPARG* gene, transcripts, and protein isoforms. In the upper part the genomic localization of* PPARG* gene is indicated, with chromosome indication, cytogenetic band, and surrounding genes. Below is depicted the exon/intron structure of* PPARG* gene with transcribed splicing variants. Transcripts encoding both the canonical and dominant negative proteins are illustrated in the left panel. The right panel shows a schematic representation of the encoded proteins with the functional domains.

Four main* PPARG* transcripts are currently known, as described by Costa et al. [3]. Additionally, our group has recently identified two isoforms acting asdominant negative toward PPAR*γ* [28], transcribed by the same promoters of PPARG2 and PPARG3 transcripts, respectively (details in Figure 1).

Using specific primers pairs (Table 1), we performed an extensive expression analysis of PPARG1, PPARG2, PPARG3, PPARG4, and ORF4 in tissues and cells related to complications of metabolic syndrome—such as altered glucose and lipids' metabolism (liver), increased inflammatory response (macrophages), atherosclerosis (EPCs, heart, and macrophages), cancer (colon carcinoma and MCF7), and thyroid dysfunction (thyroid)—and in a widely used cell model, HEK293 [3, 6, 17, 39, 40]. Given the high similarity between the 5′UTRs of PPARG1 and PPARG4 transcripts, the primers employed to analyze PPARG4 amplify both variants (distinguishable as PCR products of different size), whereas we could design PPARG1 specific primers.

**Figure 2:**
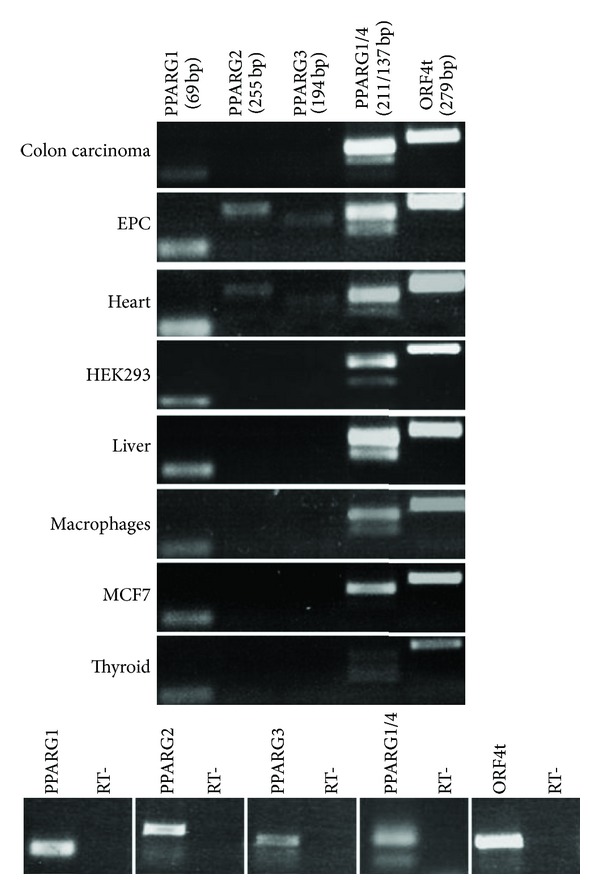
Expression pattern of* PPARG* variants in tissues and cells affected in the metabolic syndrome. For each* PPARG* transcript, specific primer pairs were used for PCR reactions. Given the similarity between PPARG1/4 5′UTRs primers amplifies both variants (distinguishable as PCR products of different sizes). “ORF4t” indicates the entire pool of ORF4 transcripts. Amplicons' sizes are shown (in bp) below transcripts' names. On the bottom panel, negative PCR controls are shown for each primer pair.

The tissue-specific expression pattern of* PPARG* alternative variants, including also transcripts encoding the same protein (PPARG1, PPARG3, and PPARG4), is shown in Figure 2. Such analysis revealed that PPARG1 transcript is expressed in all analyzed tissues and cell lines, confirming that it is abundantly and almost ubiquitously expressed in human tissues [21]. Similarly, PPARG4—which is transcribed from the same promoter—is expressed in almost all analyzed samples, albeit at lower levels than PPARG1. Therefore, we demonstrated that, in most of examined samples, PPARG1 and PPARG4 contribute to the translation of PPAR*γ*1 protein, whereas PPARG3 is expressed at low levels only in EPCs and heart. Noteworthy, also PPARG2 transcript is expressed in EPCs, as well as in the heart, whereas its expression is undetectable in other examined tissues and cell lines. This finding—possibly correlated to the anti-inflammatory role of this nuclear receptor in the cardiovascular system [41–43]—suggests that PPAR*γ*2 is predominantly expressed in these adult tissues. Surprisingly, the dominant negative isoform *γ*ORF4, till now associated with tumor pathogenesis, is expressed in all analyzed tissues and cell lines, suggesting a not negligible contribute to* PPARG* activity also in other physiologic and pathological cell processes. Of note, the results shown in Figure 2 refer to ORF4 transcripts' total expression.

### 3.2. Expression of* PPARG* Variants during Adipogenesis

**Figure 3:**
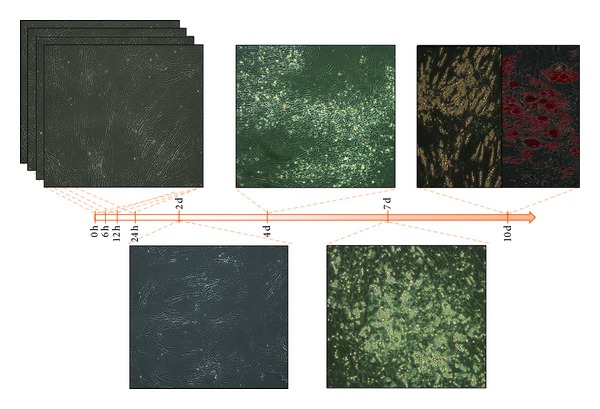
Phenotypic characteristics of undifferentiated, differentiating, and differentiated hMSCs (h = hours; d = days). Adipocyte differentiation was determined at 10 days from adipogenesis induction by Oil Red O staining of lipids vacuoles, as shown.

After* in vitro* induction of hMSCs toward adipogenic differentiation (see Methods), we selected seven different time points (Figure 3). In particular, we investigated the “early stages” of adipocyte differentiation (6, 12, and 24 hours after induction), an intermediate time point (2 days), and “late stages” (4 and 7 days) according to visible changes in cell morphology and an endpoint at 10 days when cells differentiate into adipocytes (Figure 3).

**Figure 4:**
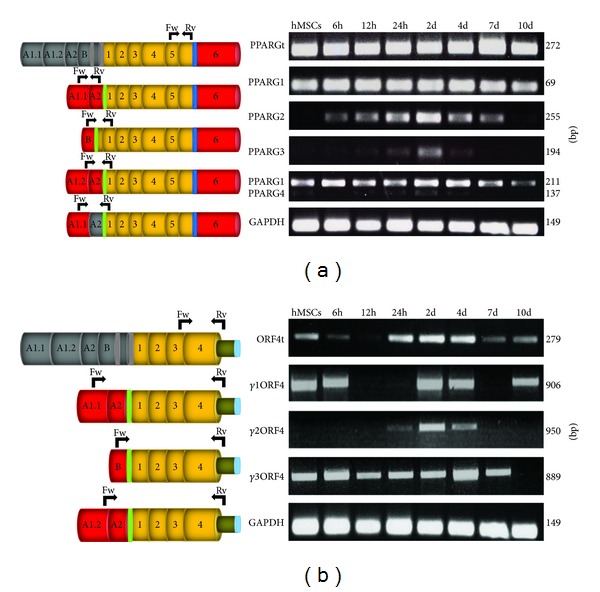
Transcript-specific RT-PCR assays for* PPARG* canonical transcripts (panel (a)) and ORF4 variants (panel (b)) at different time points of the adipogenesis (indicated on the top). On the left, the different* PPARG* transcripts are schematically shown; on the right the related PCR amplicons and their sizes (in bp) are illustrated. “PPARGt” and “ORF4t” indicate the entire pool of canonical* PPARG* and ORF4 transcripts, respectively. Transcript-specific exons are shown in grey and common exons are coloured.Black arrows indicate the specific primer pairs used in this analysis (Fw, forward; Rv, reverse).* GAPDH* was used as internal control.

RT-PCR assay revealed that, first of all, total* PPARG* expression (i.e., of the entire pool of canonical* PPARG* transcripts) is very high throughout the process. In detail, using variant-specific primers we observed that all* PPARG* transcripts are expressed—albeit at variable levels—in the examined differentiation stages (Figure 4(a)). Interestingly, PPARG2 is not expressed in hMSCs, whereas its expression is remarkably higher in the early stages after induction toward adipocyte differentiation. Particularly, as shown in Figure 4(a), this transcript reaches its highest expression after 2 days from the induction and is completely silenced at the end of the process. Similarly, PPARG3 has a mild but detectable expression only in the intermediate and late stages of cells' differentiation, with its highest expression at 2 days. This analysis revealed that PPARG1 and PPARG4 are the only canonical transcripts contributing to the final expression of PPAR*γ* protein in undifferentiated hMSCs and therefore that these cells express only PPAR*γ*1 isoform. In particular, PPARG1 is expressed at much higher levels than PPARG4 variant and, given the absence of the PPARG3 splice variant, it can be considered as the main contributor to the synthesis of functional PPAR*γ*1 protein in undifferentiated cells (Figure 4(a)).

However, PPARG1 and PPARG4 are expressed also throughout the adipocyte differentiation, although the former is the most expressed* PPARG* transcript at all the stages.

#### 3.2.1. Identification of *γ*1ORF4 and Analysis of* PPARG* Dominant Negative Transcripts

Given the existence of two different isoforms of *γ*ORF4, previously described as dominant negative of PPAR*γ* [28], we asked whether other ORF4 variants may be transcribed from the promoter upstream the A_1.1_ exon of* PPARG* gene. Thus, using specific primers pairs (described in Table 1), we were able to identify in hMSCs a novel ORF4 variant, named *γ*1ORF4 (accession number still in process; see Figure 1 for details). Similarly to PPARG1, its 5′UTR consists of A_1.1_ and A_2_ exons, whereas its coding region extends from exon 1 to 4, with a read-through in intron 4, identical to the other ORF4 transcripts (structural details in Figure 1).

Given the discovery of such new transcript in hMSCs, we decided to investigate the expression of the entire pool of ORF4 variants (ORF4t in Figure 4(b)) during* in vitro* adipogenesis. Of note, RT-PCR assay revealed that these variants are expressed along adipocytes' differentiation and particularly in the crucial stages of this process (24 hours, 2 and 4 days). Subsequent independent analysis of the three ORF4 transcripts revealed that *γ*3ORF4 variant is expressed throughout the process, whereas *γ*2ORF4 mRNA undergoes a dramatic increase at 2 days from differentiation's induction. Noteworthy, the novel variant *γ*1ORF4—identified in undifferentiated hMSCs—is expressed at variable levels during adipogenesis, although it is undetectable at some stages (Figure 4(b)).

### 3.3. Quantitative Analysis of Canonical and Dominant Negative* PPARG* Splice Variants during Adipogenesis

**Figure 5:**
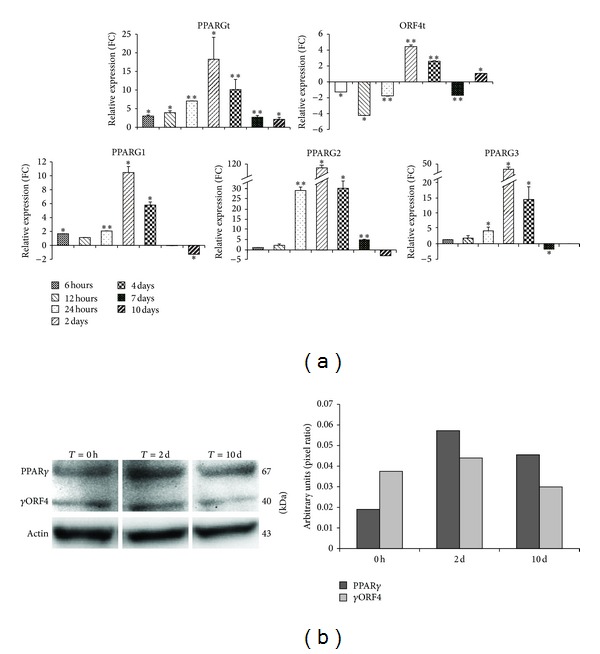
For each analyzed* PPARG* variant, bar graphs in the Panel (a) indicate the relative expression levels at different time points after i*n vitro* adipocyte differentiation. For each assay, expression is normalized for reference samples (time point at 0 or 6 hours) using* GAPDH* as housekeeping gene. Data are reported as mean values, and error bars are also reported. *P* values <0.05 are considered statistically significant and indicated by an asterisk. Double asterisks indicate *P* values <0.001. In panel (b), total cell lysates of hMSC at day 0, day 2, and day 10 by differentiation induction blotted with anti-PPAR*γ* antibody are shown. To ensure equal protein transfer, membranes were blotted with antiactin antibody. Bar graph indicates the pixel intensity ratio between PPAR*γ* isoforms and actin protein levels, reported as arbitrary units over basal (day 0).

To have a quantitative estimate of* PPARG* transcripts after induction of the adipogenic process, we performed Quantitative Real-Time analysis with specific primer pairs at the time points above described. Such quantitative analysis confirmed the findings of RT-PCR assay, showing that the expression of total* PPARG* increases up to 2 days by adipogenesis induction. Indeed, at this stage, total* PPARG* expression is about 20-fold increase compared to undifferentiated cells and it linearly decreases after 7 days, reaching expression levels comparable to undifferentiated cells (Figure 5(a)).

However, the most relevant findings derive from the canonical transcript-specific analysis. Indeed, it revealed that all PPARG canonical transcripts have a similar trend of expression but exhibit different fold increase during the process (Figure S1 see supplementary materials available online at http://dx.doi.org/10.1155/2014/537865). For PPARG2 and PPARG3 the expression values at 6 hours were used as baseline, since they are not expressed in undifferentiated hMSCs (Figure 5(a)). However, despite their low expression levels, these transcripts exhibit an increase of expression considerably higher than PPARG1. Indeed, at 2 days by differentiations' induction, the expression of PPARG2 and PPARG3 raises of about 110- and 45-fold, respectively, whereas PPARG1 increase is of about 10-fold (Figure 5(a)).

To quantitatively study ORF4 transcripts, the only way to discriminate among the different variants is through the analysis of large PCR amplicons (about 900–1000 bp, Figure 4(b)), unfeasible with qRT-PCR. Thus, quantitative data for ORF4, shown in Figure 5(a), refer to the pool of ORF4 transcripts. Particularly, we observed, for these variants, a different trend of expression throughout the process compared to* PPARG* canonical transcripts, confirming RT-PCR assays (Figure 4(b)). Indeed, ORF4 total expression is significantly downregulated in early stages of differentiation and reaches its highest values at 2 days. Nonetheless, its increase is considerably lower than the canonical transcripts (fold increase = 4; Figure 5(a)).

Finally, pairwise comparison of fold changes' variation, that is, between two subsequent time points, revealed that the most significant increase of the expression values occurs in the transition from day 1 to day 2 upon induction of adipocyte differentiation (Figure S1). Notably, the most striking increase has been observed for PPARG2 and PPARG3 variants (about 90 and 40 fold, resp.), suggesting the inducible nature of their promoters during this process. On the opposite, highly significant decreases were observed—for these two splice variants—immediately after day 2 from the induction of the process. A common behavior was observed for PPARG1 and ORF4 transcripts. In particular, these variants undergo mild expression changes in the transitions among the stages, showing a quite constant basal expression throughout the adipogenic process (Figure 5(a) and S1). Since the most evident changes in* PPARG* transcripts' abundance were detected after 2 days by differentiation induction, we investigated protein levels on three time points, day 0 (undifferentiated cells), day 2 (i.e., the highest peak of* PPARG* expression), and day 10 (i.e., differentiated cells). As no commercially available antibodies exist for ORF4 protein, we used a polyclonal antibody directed against the N-terminal domain, able to recognize both the canonical and the shortest* PPARG* isoforms. We detected canonical PPAR*γ* at 67 kDa and immunoreactive bands at 40 kDa, the predicted weight of ORF4 protein isoform. As expected, consistently with the changes in mRNA levels, after 2 days by differentiation induction, the expressions of PPAR*γ*—and of the shortest isoforms—were higher compared to both undifferentiated and completely differentiated cells (Figure 5(b)).

## 4. Conclusions 

Epidemiological studies demonstrate that the prevalence of the metabolic syndrome is increasing in the Western world and developing countries, and to date an adequate therapy has not been yet established [17, 44].

Undoubtedly,* PPARG* is one of the most studied genes accounting for metabolic disorders. Indeed, it modulates the expression of several genes with a crucial role in glucose, lipid and cholesterol metabolism, insulin signaling, and adipokines' production, whose imbalance leads to insulin resistance, obesity, type 2 diabetes, and cardiovascular diseases [3, 4, 7, 14]. PPAR*γ* is also a drug target and, currently, its synthetic ligands are used to treat hyperlipidemia and as insulin-sensitizing antidiabetic agents [18]. Thus, defining* PPARG* activity in tissues and cells related to energy metabolism may provide useful insights to develop new and effective therapeutic strategies to treat the metabolic syndrome and its complications.

It is currently known that—by different promoter usage and alternative splicing—the human* PPARG* gene generates multiple variants encoding two proteins, PPAR*γ*1 and PPAR*γ*2. Since different* PPARG* splice variants encode the same protein isoform, their differential expression, both spatial and temporal, may reflect a different regulation, translation, mRNA stability, and/or localization. To complicate the picture, the recent identification of *γ*ORF4 isoform—able to act as dominant-negative and with a tumorigenic effect [28]—suggests that PPAR*γ* activity is modulated through transcript-specific regulation.

Therefore, our effort has been to investigate* PPARG* expression in different tissues and cells—affected in metabolic syndrome—and during hMSCs' adipocyte differentiation. Other than focusing on canonical* PPARG* transcripts, a particular emphasis was posed toward defining the expression pattern of its variants encoding dominant negative isoforms. In our study we identified *γ*1ORF4, a novel* PPARG* transcript that, similarly to the previously described *γ*2ORF4 and *γ*3ORF4 [28], may act as dominant negative toward PPAR*γ*.

Our expression analysis has clearly demonstrated that the different promoters of* PPARG* have a peculiar transcriptional activity. Such finding is particularly relevant in the adipocyte differentiation, in which PPAR*γ* is a key player [4, 9, 29]. The almost ubiquitous PPARG1/PPARG4 expression, particularly throughout adipogenesis, indicates a more pronounced activity of their promoter compared to the others, suggesting it as the main contributor to PPAR*γ* protein synthesis. Furthermore, the mild expression changes of PPARG1 along adipocyte differentiation strengthen the hypothesis that its promoter provides constitutive levels of* PPARG* messengers. On the opposite, the tissue- and stage-specific PPARG2 expression, as well as its dramatic variations throughout the adipogenesis, clearly demonstrate its inducible nature.

Interestingly, the almost ubiquitous expressions of ORF4 variants in tissues and cells, as well as during adipogenesis, support the hypothesis that* PPARG* regulates itself through dominant negative isoforms. Furthermore, our results suggest that similarly to* PPARG* canonical transcripts, the three ORF4 variants give a different contribution to PPAR*γ* activity. Indeed, whereas *γ*1ORF4 and *γ*2ORF4 exhibit stage-specific expression, *γ*3ORF4 is constantly expressed along adipocyte differentiation but not in mature adipose cells. These findings, strictly correlated with those regarding the canonical isoforms, suggest that (1) the promoter upstream exon B is inducible for both the canonical and ORF4 variants, (2) constitutive levels of* PPARG* variants, encoding dominant negative isoforms, are provided throughout differentiation by the promoter upstream noncoding exon A_1.2_, and (3) it almost exclusively transcribes *γ*3ORF4 rather than the canonical PPARG3. Therefore, such evidences suggest a relevant—if not exclusive—role of promoter of *γ*3ORF4 and PPARG3 variants in negative PPAR*γ* regulation. In addition, protein analysis confirmed that after 2 days by differentiation induction PPAR*γ* protein has a higher expression compared to undifferentiated and completely differentiated cells. Moreover, we observed the same trend of expression also for a shorter protein of 40 kDa, corresponding to the predicted weight of ORF4 isoform.

Although the results described herein represent only a starting point to understand the impact of* PPARG* transcripts along human adipogenesis, they support the notion that this generegulates such crucial process through balancing the levels of its different splicing variants. Further studies— particularly taking into account* PPARG* protein products—are strictly required to definitely establish the role of all splicing variants in adipocyte differentiation. Notably, our results shed light on previously underestimated aspects of* PPARG* regulation and propose a yet unexplored role of its dominant negative isoforms during adipogenesis. Indeed, the finding that—during a crucial process in which* PPARG* is a “master gene”—both the transcripts and the proteins encoding dominant negative isoforms are constitutively expressed and/or can be modulated similarly to the canonical* PPARG* variants, enforces the need to investigate toward this direction. Understanding more about* PPARG* activity in the adipogenic process is directly linked to its possible contribution to the onset and progression of metabolism-related pathologies, including the metabolic syndrome and its complications.

Finally, we cannot exclude that the presence of transcripts encoding* PPARG* dominant negative proteins in other human tissues may underlie their interesting roles in physiological processes as well as in other pathological conditions.

## Supplementary Material

Pairwise comparison of fold change variations for PPARG transcripts between two subsequent time points in hMSCs' differentiation into adipocytes.

## Figures and Tables

**Table 1:** Primer pairs for canonical and dominant negative *PPARG* variants.

Transcript	Oligonucleotide pairs		Size (bp)
	Forward	Reverse	
tPPARG	GAGAAGGAGAAGCTGTTGGC	ATGGCCACCTCTTTGCTCT	272
PPARG1	CGAGGACACCGGAGAGGG	TGTGGTTTAGTGTTGGCTTCTT	69
PPARG2	TTTTAACGGATTGATCTTTTGC	AGGAGTGGGAGTGGTCTTCC	255
PPARG3	TTCTGCTTAATTCCCTTTCC	AGGAGTGGGAGTGGTCTTCC	194
PPARG1/4	CGAGGACACCGGAGAGGG	AGGAGTGGGAGTGGTCTTCC	211/137
tORF4	CTTGCAGTGGGGATGTCTCA	AAACCCAAAACAACTTCCCG	279
*γ*1ORF4	CGAGGACACCGGAGAGGG	AAACCCAAAACAACTTCCCG	906
*γ*2ORF4	TTTTAACGGATTGATCTTTTG	AAACCCAAAACAACTTCCCG	950
*γ*3ORF4	TTCTGCTTAATTCCCTTTCC	AAACCCAAAACAACTTCCCG	889
