# Supplementary material for: PPARG in Human Adipogenesis: Differential Contribution of Canonical Transcripts and Dominant Negative Isoforms
Source: PPAR Res. 2014 Mar 23;2014:537865. doi: 10.1155/2014/537865 (PMC3981527; doi:10.1155/2014/537865)
Supplement: Supplementary file 1 — Pairwise comparison of fold change variations for PPARG transcripts between two subsequent time points in hMSCs' differentiation into adipocytes. [file 537865.f1.pdf]

Supplementary File 1.

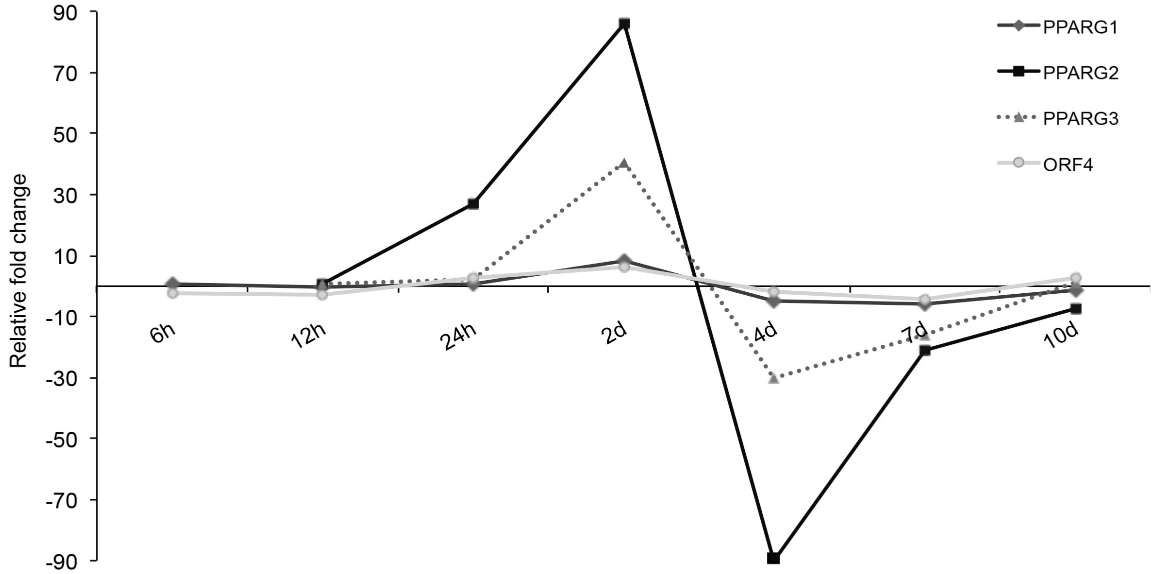

Figure legend Supplementary File 1.

Pairwise comparison of fold change variations for PPARG transcripts between two subsequent time points in hMSCs' differentiation into adipocytes.
